# Augmented trophoblast cell death in preeclampsia can proceed via ceramide-mediated necroptosis

**DOI:** 10.1038/cddis.2016.483

**Published:** 2017-02-02

**Authors:** Liane Jennifer Bailey, Sruthi Alahari, Andrea Tagliaferro, Martin Post, Isabella Caniggia

**Affiliations:** 1 Lunenfeld-Tanenbaum Research Institute, Mount Sinai Hospital, Toronto, Ontario, Canada; 2 Institute of Medical Science, University of Toronto, Toronto, Ontario, Canada; 3 Department of Physiology, University of Toronto, Toronto, Ontario, Canada; 4 Program in Physiology and Experimental Medicine, Peter Gilgan Centre for Research and Learning, Hospital for Sick Children, Toronto, Ontario, Canada; 5 Department of Obstetrics and Gynecology, University of Toronto, Toronto, Ontario, Canada

## Abstract

Preeclampsia, a serious hypertensive disorder of pregnancy, is characterized by elevated ceramide (CER) content that is responsible for heightened trophoblast cell death rates via apoptosis and autophagy. Whether trophoblast cells undergo necroptosis, a newly characterized form of regulated necrosis, and the potential role of CER in this process remain to be established. Herein, we report that exposure of both JEG3 cells and primary isolated cytotrophoblasts to C16:0 CER in conjunction with a caspase-8 inhibitor (Q-VD-OPh) promoted necroptotic cell death, as evidenced by increased expression and association of receptor-interacting protein kinases RIP1 and RIP3, as well as phosphorylation of mixed lineage kinase domain-like (MLKL) protein. MLKL activation and oligomerization could be abrogated by pretreatment with the necroptosis inhibitor necrostatin-1 (Nec-1). CER+Q-VD-OPH-treated primary trophoblasts displayed striking necrotic morphology along with disrupted fusion processes as evidenced by maintenance of E-cadherin-stained membrane boundaries and reduced glial cell missing-1 expression, but these events were effectively reversed using Nec-1. Of clinical relevance, we established an increased susceptibility to necroptotic cell death in preeclamptic placentae relative to normotensive controls. In preeclampsia, increased necrosome (RIP1/RIP3) protein levels, as well as MLKL activation and oligomerization associated with necrotic cytotrophoblast morphology. In addition, caspase-8 activity was reduced in severe early-onset preeclampsia cases. This study is the first to report that trophoblast cells undergo CER-induced necroptotic cell death, thereby contributing to the increased placental dysfunction and cell death found in preeclampsia.

Necroptosis is a mode of cell death that has overturned the traditional view of necrosis as merely a passive process. Although necroptosis shares the same morphological features as necrosis, such as organelle swelling and plasma membrane rupture, it occurs as a result of tightly regulated caspase-independent signaling events.^1^ Necroptosis is typically initiated by stimuli such as tumor necrosis factor (TNF), Fas ligand or TNF-related apoptosis-inducing ligand (TRAIL) binding to their respective death domain-containing receptor at the cell membrane, leading to activation of receptor-interacting protein kinase 1 (RIP1).^2^ Under apoptotic conditions, active caspase-8 prevents further necroptotic signaling by cleaving and inactivating RIP1 and its kinase partner named receptor-interacting protein kinase 3 (RIP3), ultimately initiating apoptosis.^3, 4^ Conversely, under conditions of caspase-8 inhibition, RIP1 recruits RIP3 to the necrosome complex where they participate in phosphorylation events required for its stabilization.^5^ In particular, RIP3 phosphorylates the mixed lineage kinase domain-like protein (MLKL),^6^ thus permitting its oligomerization and subsequent translocation to the plasma membrane.^7^ Phosphorylated MLKL (p-MLKL) carries out the final execution of necroptotic cell death by permeabilizing the cell membrane^8, 9, 10^ Necroptosis has been implicated in a variety of vascular and inflammatory disorders and inhibition of necroptosis has been shown to improve health outcomes in several animal models of human diseases^11^

Preeclampsia is a serious hypertensive disorder that occurs in 5–7% of pregnancies, and represents a leading cause of maternal and perinatal morbidity and mortality worldwide. Although the exact pathophysiology of this disease is not fully understood, it is established that the placenta is critical for the onset of the clinical manifestation.^12^ The only current way to resolve this disorder is delivery, which may expose the newborn to short- and long-term health risks related to prematurity. Placentae from preeclamptic women are characterized by excessive cell death, including apoptosis^13, 14^ and autophagy.^15, 16, 17^ However, the involvement of necroptosis in placental cell death remains unknown.

We have recently identified that disruption of sphingolipid metabolism leading to ceramide (CER) accumulation is responsible for increased trophoblast cell death in preeclampsia.^16^ CER has a critical role in cell death, particularly in the initiation of apoptosis and autophagy.^18, 19^ In this study, we sought to examine whether trophoblast cells undergo necroptosis under physiological and pathological conditions of pregnancy, and whether CER acts as a mediator of this process. Our findings show that C16:0 CER treatment particularly under conditions of caspase inhibition promotes necroptotic cell death in trophoblastic JEG3 cells and primary isolated cytotrophoblast cells. Furthermore, preeclamptic placentae exhibit higher rates of necroptosis compared with normotensive controls, and we propose that this results from the high CER levels and reduced caspase-8 activity present in these placentae.

## Results

### CER stimulates the necroptotic machinery

**Figure 1:**
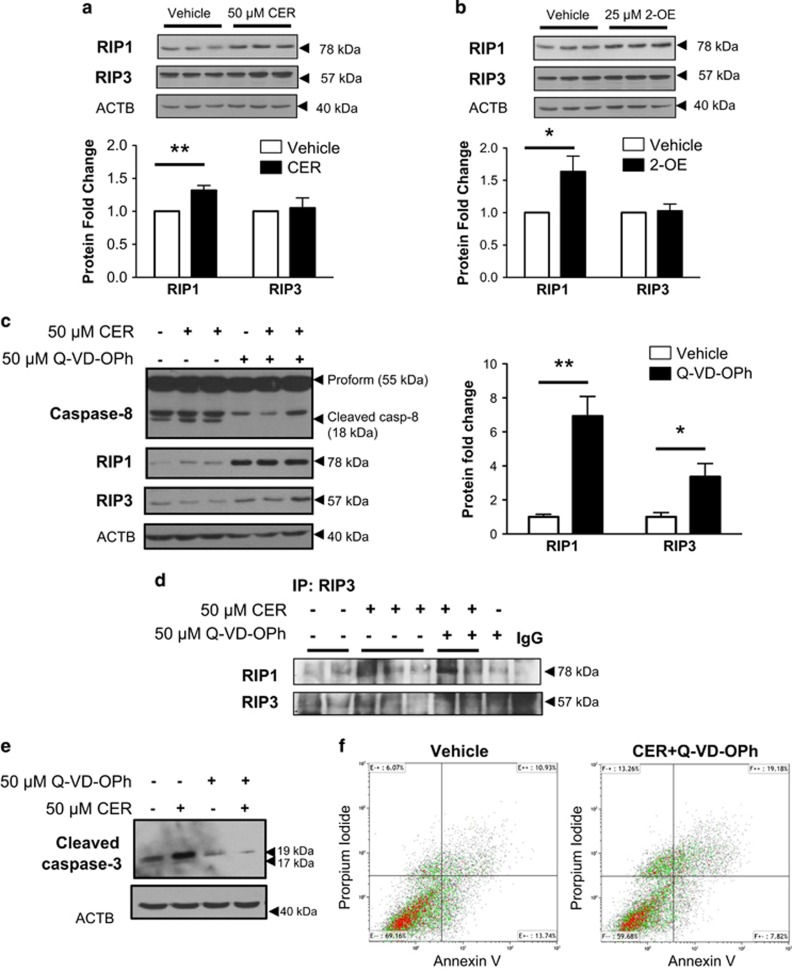
CER stimulates the necrosome in JEG3 cells. (**a**) Representative western blots and densitometric analysis of RIP1 and RIP3 kinases in choriocarcinoma JEG3 cells exposed to 50 *μ*M C16:0 CER for 24 h. RIP3 protein levels were normalized to *β*-actin (ACTB) and expressed as fold change relative to vehicle control (EtOH). (**b**) Representative western blots and densitometric analysis for RIP1 and RIP3 kinases after exposure of JEG3 cells to 25 *μ*M of 2-OE for 24 h. Values were normalized to ACTB and expressed as a fold change relative to vehicle control (DMSO). (**a** and **b**) Statistical significance was determined as *P<0.05 using an unpaired Student's *t*-test (*n*=5 different experiments carried out in triplicate). (**c**) Representative western blots for caspase-8, RIP1 and RIP3 following exposure of JEG3 cells to the pan-caspase inhibitor Q-VD-OPh (50 *μ*M). Densitometric quantification of RIP1 and RIP3 levels following Q-VD-OPh treatment normalized to ACTB and expressed as a fold change relative to DMSO vehicle control. Statistical significance was determined as *P<0.05 or **P<0.01 using an unpaired Student's *t*-test (*n*=4 separate experiments, carried out in duplicate). (**d**) Immunoprecipitation (IP) of RIP3 followed by WB for RIP1/RIP3 in JEG3 cells treated with 50 *μ*M CER alone or CER+Q-VD-OPh relative to vehicle control or 50 *μ*M Q-VD-OPh alone (*n*=3 separate experiments). Goat IgG was used as a negative control. (**e**) Representative western blot of cleaved caspase-3 following exposure of JEG3 cells to 50 *μ*M CER±50 *μ*M Q-VD-OPh (*n*=3). ACTB was used as a loading control. (**f**) JEG3 cells treated with CER+Q-VD-OPh analyzed by flow cytometry Annexin V and propidium iodide double-labeling display an increase in necrotic cells (propidium iodide^+^) accompanied by a reduction of early apoptotic cells (Annexin V+/propidiumiodide^−^) compared with vehicle control cells. (*n*=3)

Excessive trophoblast cell death as a result of CER accumulation contributes to the placental dysfunction typical of preeclampsia.^16^ Hence, we examined the effect of CER on necrosome protein expression in human choriocarcinoma JEG3 cells, an established *in vitro* model of placental origin. Exposure of JEG3 cells to 50 *μ*M synthetic C16:0 CER for 24 h significantly increased RIP1 protein levels as assessed by western blot analysis (Figure 1a). In contrast, no changes in RIP3 kinase expression were found following C16:0 CER treatment (Figure 1a). Exposure of JEG3 cells to the ceramidase inhibitor 2-oleoylethanolamine (2-OE) resulted in increased RIP1 levels but did not affect RIP3 expression (Figure 1b), similarly to that found with C16:0 CER treatment.

Necroptosis is a cell death process that can only occur in the absence of active caspase-8. Quinolyl-valyl-O-methylaspartyl-[2,6-difluorophenoxy]-methyl-ketone (Q-VD-OPh) is a potent inhibitor of caspase activity and apoptosis that has been extensively used for investigating necroptosis *in vitro*.^8, 20^ Exposure of JEG3 to 50 *μ*M of Q-VD-OPh resulted in abrogation of caspase-8 cleavage from its 55 kDa proform to its 18 kDa active fragment (Figure 1c, upper panel). Furthermore, treatment of JEG3 cells with Q-VD-OPh in the presence or absence of C16:0 CER exhibited significant increases in RIP1 and RIP3 protein levels when compared with vehicle DMSO control treatment (Figure 1c), supporting the notion that inhibition of caspase-8 cleavage is critical to prime the necroptotic machinery in JEG3 cells. As RIP1 binding to RIP3 is a prerequisite for necrosome activation, we next assessed the association of these kinases. Immunoprecipitation of RIP3 followed by western blotting for RIP1 revealed an increased RIP1/RIP3 association in cells exposed to CER and CER+Q-VD-OPh, which is indicative of necrosome assembly (Figure 1d). Exposure of JEG3 cells to Q-VD-OPh was found to block cleaved caspase-3 (Figure 1e) indicating abrogation of apoptotic cell death in favor of necroptosis. Similarly, flow cytometry analysis revealed increased necroptotic cell death in JEG3 cell upon CER+Q-VD-OPh exposure as identified by a twofold increase of Annexin V^−^/propidium iodide^+^ (indicative of primary necrosis) or Annexin V^+^/propidium iodide^+^ cells (secondary necrosis), and a twofold decrease of Annexin V^+^/propidium iodide^−^ cells that is characteristic of apoptosis (Figure 1f).

**Figure 2:**
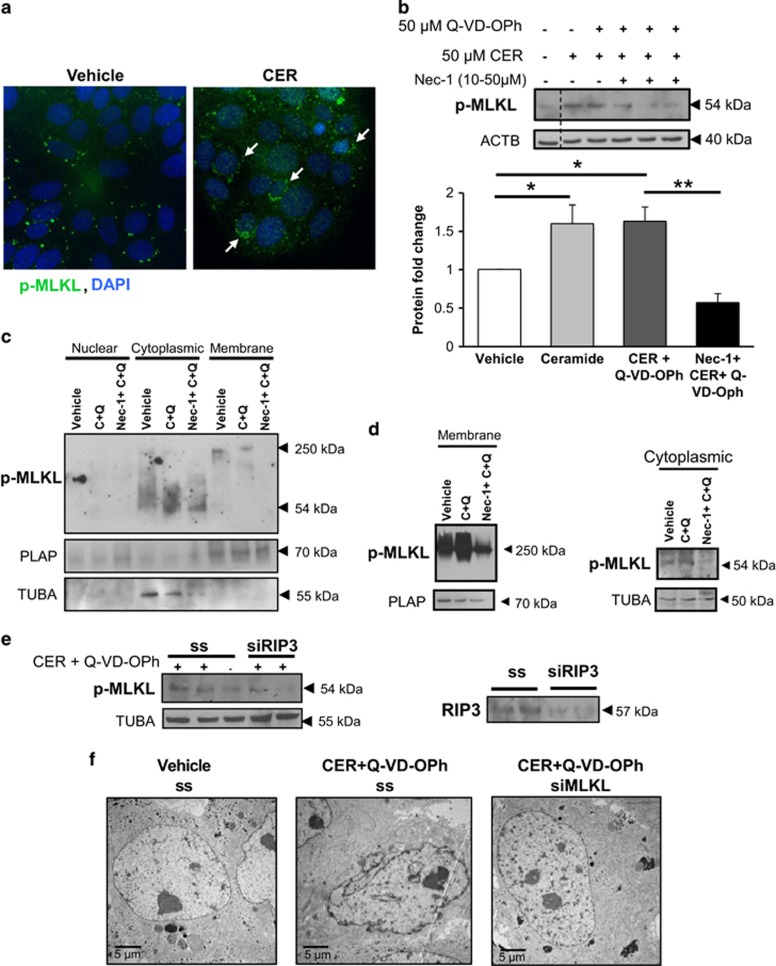
CER and Q-VD-OPh induce activation of MLKL in JEG3 cells. (**a**) Immunofluorescence analysis showed increased cytoplasmic signal for p-MLKL in JEG3 cells following exposure to C16:0 CER relative to control vehicle (*n*=3 separate experiments, performed in duplicate). p-MLKL appeared as punctate signal that clustered in the cytoplasm (white arrows), particularly in CER-treated cells. p-MLKL (green) and nuclei counterstained with DAPI (blue). (**b**) Treatment with 50 *μ*M CER alone with and without 50 *μ*M Q-VD-OPh (CER+Q-VD-OPh) resulted in phosphorylation of MLKL to its active form (*n*=5 different experiments). Nec-1 pretreatment (25 *μ*M) led to a significant protection of JEG3 cells from CER+Q-VD-OPh-induced MLKL activation, as measured by p-MLKL expression (*n*=4 different experiments). Protein levels were normalized to ACTB and densitometric quantification is expressed as fold change relative to vehicle control (EtOH+DMSO). Statistical significance was determined as *P<0.05 or **P<0.01 using one-way ANOVA with *post-hoc* Tukey's test. (**c**) Native polyacrylamide gel electrophoresis (PAGE) analysis of membrane and cytoplasmic fractions isolated from JEG3 cells treated with CER and Q-VD-OPh, or following Nec-1 pretreatment. Western blot analysis showed p-MLKL expression as an oligomer of high molecular weight (250 kDa) in the membrane-enriched fraction, as well as a monomer (54 kDa) in the cytoplasmic fraction. Nec-1 protected JEG3 cells from CER+Q-VD-OPh-induced MLKL activation and oligomerization. Placental alkaline phosphatase (PLAP) was used as the loading control for membrane fractions, whereas TUBA was used as the cytoplasmic loading control. (**d**) Native PAGE analysis of p-MLKL from membrane and cytoplasmic fractions isolated by Triton X-114 phase separation from JEG3 cells treated with CER+QVDOPh, and Nec-1 pretreatment. PLAP was used as the loading control for membrane fractions, and alpha-tubulin (TUBA) was used as a cytoplasmic marker. (**e**) Western blot analysis showed that siRNA treatment against MLKL reduced its protein expression in JEG3 cells compared with cells treated with a scramble sequence (ss). Alpha-tubulin (TUBA) was used as a loading control. (**f**) TEM of CER+Q-VD-OPh-treated JEG3 (with scramble sequence) display necrotic alterations in cellular and nuclear morphology compared with vehicle controls, but these changes were abrogated with siRNA knockdown of MLKL

MLKL is a key regulator of necroptosis, as it functions as the final executor of necroptotic cell death downstream from activated RIP1 and RIP3.^10^ Immunofluorescence staining for p-MLKL in JEG3 cells demonstrated augmented p-MLKL signal following C16:0 CER treatment (Figure 2a). p-MLKL exhibited a punctate appearance and presence of cytoplasmic clusters that were prominent in CER-treated cells compared with controls (Figure 2a). The specificity of p-MLKL antibody was validated using a blocking peptide that efficiently competed for the predicted 54-kDa product (Supplementary S1a). In line with our immunofluorescence findings, western blotting of JEG3 cells treated with CER alone or in combination with Q-VD-OPh showed significantly higher levels of p-MLKL, the active form of the protein, relative to controls (Figure 2b). Importantly, pretreatment of JEG3 cells with the necroptosis inhibitor necrostatin-1 (Nec-1) prevented the CER+Q-VD-OPh-induced MLKL phosphorylation (Figure 2b). As Nec-1 acts by inhibiting RIP1 kinase activity, these data demonstrate that in response to CER accumulation and caspase inhibition, trophoblasts proceed to cell death via a RIP1-dependent regulated necroptosis.

The presence of cytoplasmic clusters of p-MLKL (Figure 2a) is indicative of its oligomerization, and activated MLKL is known to oligomerize and induce necroptotic cell death. To further establish the formation of MLKL oligomers, we induced necroptosis in JEG3 cells with CER+Q-VD-OPh hindrance, isolated the membrane fraction and ran the samples under non-reducing conditions. Western blot analysis revealed a protein molecular weight shift to a band of approximately 250 kDa, likely corresponding to MLKL oligomers (Figure 2c). Moreover, in line with our previous western blot findings using reducing conditions, a monomeric 54-kDa form of p-MLKL was detected exclusively in the cytoplasmic fraction and was increased following CER+Q-VD-OPh treatment. Importantly, Nec-1 treatment abolished p-MLKL expression (monomer and oligomers) in both the cytoplasmic and membrane fractions relative of CER+Q-VD-OPh-treated cells (Figure 2c). PLAP and *α*-tubulin (TUBA) antibodies were used to determine the purity of membrane and cytoplasmic fractions, respectively. These findings were confirmed using a temperature sensitive phase separation to isolate membrane and cytoplasmic proteins (Figure 2d). Thus, by inhibiting RIP1 kinase activity, Nec-1 can prevent MLKL phosphorylation and oligomerization induced by CER and caspase inhibition. Our results were further corroborated by RIP3 knockdown using short interfering RNA (siRNA) that prevented the CER+Q-VD-OPh stimulatory effect on p-MLKL expression in JEG3 cells when compared with control scrambled-treated cells in the presence of CER+Q-VD-OPh (Figure 2e). Similarly, MLKL siRNA conferred protection of JEG3 cells from CER-induced necroptosis as shown by maintenance of nuclear shape, cytoplasmic density and intact cell boundaries compared with control scrambled-treated cells exposed to CER+Q-VD-OPh in transmission electron microscopy (TEM) images (Figure 2f).

### Preeclamptic placentae have increased susceptibility to necroptosis

**Figure 3:**
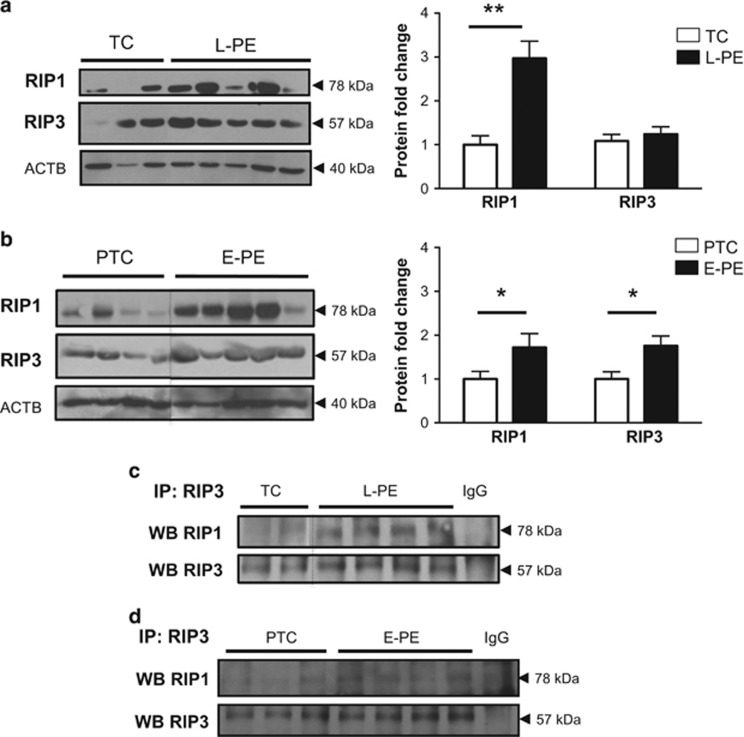
RIP1 and RIP3 expression and association in preeclamptic placentae. (**a**) RIP1 protein expression was significantly increased in late-onset preeclamptic (L-PE) placentae relative to TCs as detected by western blotting (WB). No significant changes were detected for RIP3. Densitometric analysis of RIP1 and RIP3 expression in L-PE placentae normalized to ACTB (TC: *n*=12, L-PE: *n*=20). Statistical significance was determined as **P<0.01 using an unpaired Student's *t*-test. (**b**) WBs of RIP1 and RIP3 expression showed increased levels of both proteins in early-onset preeclampsia (E-PE) placentae relative to preterm controls (PTC). Densitometric analysis of RIP1 and RIP3 expression in E-PE normalized to ACTB (PTC: *n*=12, E-PE: *n*=22). Statistical significance was determined as *P<0.05 using an unpaired Student's *t*-test. (**c** and **d**) Immunoprecipitation (IP) of RIP3 followed by WB of RIP1/RIP3 showed enhanced RIP1/RIP3 association in E-PE and L-PE relative to PTC (PTC *n*=7; E-PE: *n*=8; L-PE: *n*=9; TC *n*=7). Goat IgG was used as a negative control

**Figure 4:**
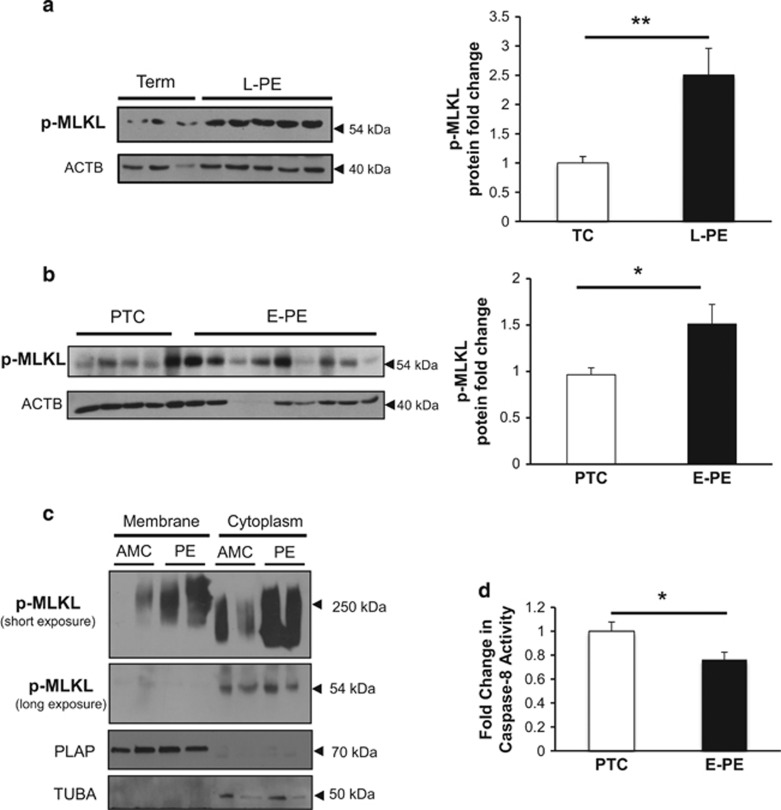
MLKL activation in preeclampsia associates with reduced caspase-8 activity. (**a** and **b**) p-MLKL protein expression in L-PE and E-PE placentae relative to TC and PTC controls as detected by western blotting. Densitometric analysis of p-MLKL expression in L-PE and E-PE placentae normalized to ACTB (L-PE, *n*=22; TC, *n*=10; E-PE, *n*=20; PTC, *n*=10). Statistical significance was determined as **P<0.01 using an unpaired Student's *t*-test. (**c**) Native PAGE analysis of membrane isolated from preeclamptic placentae and age-matched controls (AMC). Representative western blots demonstrated p-MLKL expression as an oligomer of high molecular weight (250 kDa) as well as a monomer of 54 kDa in the membrane and cytoplasmic fractions, respectively. PLAP represents the loading control for the membrane fractions, whereas TUBA was used as the cytoplasmic loading control. (**d**) Caspase-8 activity assay in placentae from E-PE and PTC (E-PE, *n*=6; PTC, *n*=6)

The ACOG guidelines define preeclampsia as a spectrum of disorders, with early-onset (E-PE) and late-onset preeclampsia (L-PE) cases categorized as different subtypes.^21^ Hence, we first mapped the key protein factors of necroptosis in E-PE, L-PE and normotensive age-matched preterm (PTC) and term control (TC) placentae. Western blot analysis demonstrated significantly increased RIP1 protein expression levels in tissue lysates from both E-PE and L-PE when compared with controls (Figures 3a and b). Although no changes were observed in RIP3 expression between L-PE and TC groups, RIP3 levels were significantly higher in E-PE compared with PTC (Figures 3a and b). Binding of RIP1 to RIP3 was increased in tissue from both E-PE and L-PE placentae relative to controls, as shown by immunoprecipitation studies (Figures 3c and d) suggesting increased necrosome formation. In addition, activated p-MLKL levels were significantly increased in both L-PE and E-PE placentae relative to controls (Figures 4a and b). Of note, mode of delivery, labor and fetal sex did not affect placental RIP1 and p-MLKL levels (Supplementary Figure S2).

In order to further investigate whether necroptosis is active in the human placenta, we performed cell membrane isolations in placental tissue from preeclamptic pregnancies and controls. As previously observed in JEG3 cells following CER+Q-VD-OPh hindrance, membrane fraction samples under non-reducing conditions demonstrated a p-MLKL band at 250 kDa representing MLKL oligomers in the membrane fraction, which was markedly increased in preeclamptic samples (Figure 4c, upper panel). The cytoplasmic fraction also presented a high molecular weight band shifted to approximately 200 kDa, which may reflect an incompletely assembled and MLKL oligomer (Figure 4c, upper panel). Furthermore, the monomeric form of p-MLKL was restricted to the cytoplasmic compartment and was slightly increased in preeclampsia relative to controls (Figure 4c, second panel). These data provide evidence for MLKL activation and oligomerization at cell membrane in preeclamptic placentae.

As blockage of caspase-8 function is required for shunting cell death toward necroptosis, we next examined caspase-8 activity. Although caspase-8 activity was significantly decreased in E-PE tissue relative to PTC (Figure 4d), no changes were found in L-PE (data not shown).

**Figure 5:**
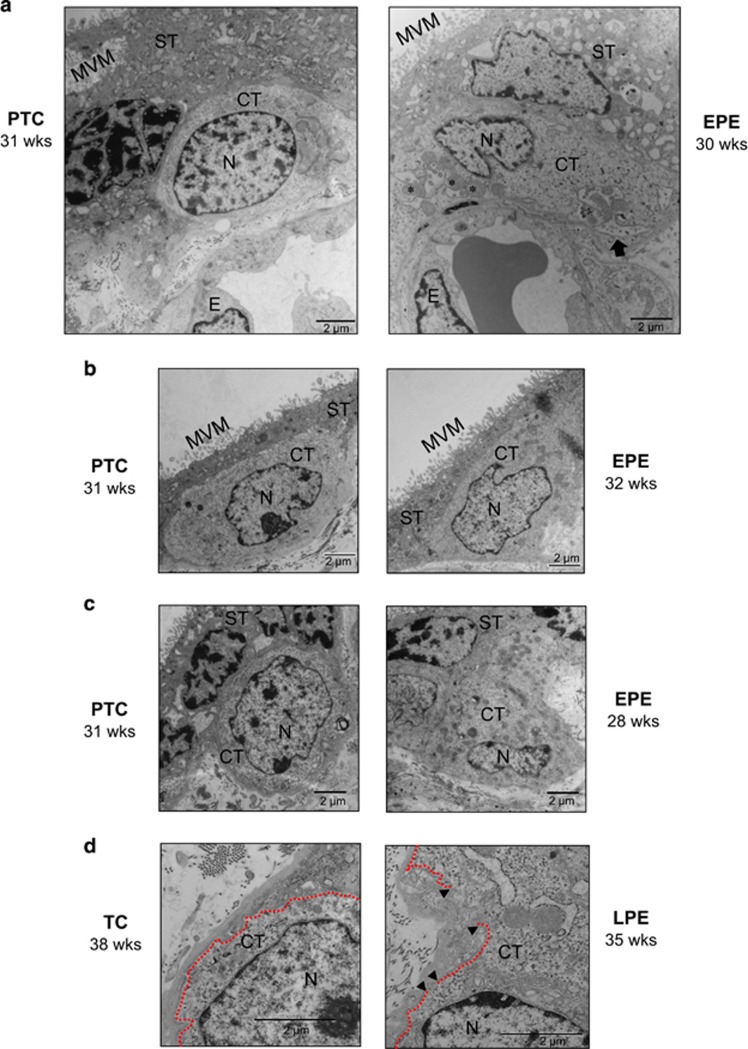
Preeclampsia is associated with necroptotic cytotrophoblast cell morphology. (**a**) TEM analysis of PTC and PE placental tissue sections. Necrosis morphology was identified based on swollen mitochondria (indicated by asterisks), loss of cytoplasmic electron density, and membrane disruptions. An arrow designates evidence of mitophagy in the PE sample. (**b** and **c**) Representative TEM images of cytotrophoblast cells from E-PE and PTC placentae, showing loss of chromatin organization (chromatin decondensation), aberrant nuclear morphology and loss of cytoplasmic electron density in PE samples compared with PTC. (**d**) Representative TEM images from L-PE and TC placentae showing discontinuous cell membrane of necrotic cytotrophoblasts from PE placentae, as occurs in the final stages of necrosis. This is depicted by gaps (indicated by black arrowheads) in the plasma membrane (depicted with dashed line). CT, cytotrophoblast; E, endothelial cell; MVM, microvillous membrane; N, nucleus; ST, syncytiotrophoblast. Scale bars=2 *μ*m

We further examined placental tissue by TEM analysis to confirm the presence of necroptotic events *in vivo*. Placental tissue from normotensive controls exhibited cytotrophoblasts with a smooth nuclear envelope and a normally shaped nucleus containing reticulated nucleoli, healthy cytoplasmic density and organelles, along with clearly outlined cell boundaries (Figures 5a–d, left panels). In stark contrast, a subset of cytotrophoblasts from preeclamptic cases showed early signs of necroptosis, including swollen mitochondria (Figure 5a, right panel), loss of cytoplasmic density and aberrant nuclear shape without chromatin condensation (Figures 5b and c, right panels), and a discontinuous cell membrane (Figure 5d, left panel). These necrotic features were prevalent in both E-PE and L-PE placentae; however, they were mainly observed in cytotrophoblast cells, but not in syncytiotrophoblasts, suggesting that this cell death is restricted to the cytotrophoblast layer.

### Nec-1 protects cytotrophoblasts from CER-induced necroptotic cell death

**Figure 6:**
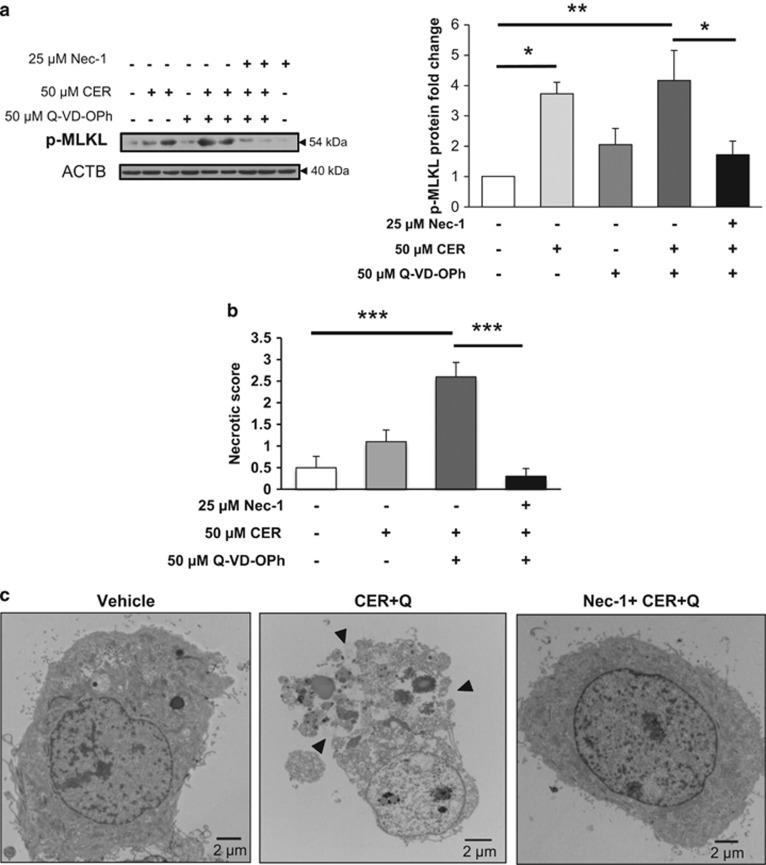
Nec-1 protects primary trophoblast cells from CER+Q-VD-OPh-induced necroptosis. (**a**) Treatment with 50 *μ*M CER alone (*P<0.05) or in combination with 50 *μ*M Q-VD-OPh (CER+Q-VD-OPh, **P<0.01) resulted in significant phosphorylation of MLKL. In all, 25 *μ*M Nec-1 pretreatment led to a significant protection of primary isolated cytrophoblast cells from CER+Q-VD-OPh-induced MLKL phosphorylation (*P<0.05, *n*=5 independent cell culture isolations). Protein levels were normalized to ACTB and expressed as fold change relative to vehicle control (EtOH+DMSO). Statistical significance was determined as *P<0.05 or **P<0.01 using one-way ANOVA with *post-hoc* Tukey's test. (**b**) Sum of the four necrotic morphological criteria (cytoplasm translucence, loss of plasma membrane integrity, swollen mitochondria and decondensed chromatin) quantified as an overall necrotic score in CER+Q-VD-OPh-treated cells relative to vehicle control cells. CER+Q-VD-OPh causes striking necrotic morphology, while 1-h pretreatment with Nec-1 protects against these necrotic changes. The image scoring test was found to be highly reliable. (*α*=0.904, 200 items) and statistical significance was determined as ***P<0.001 using one-way ANOVA with *post-hoc* Tukey's test. (**c**) Representative TEM images of primary isolated trophoblasts showed necrotic cell membrane rupture and cytoplasmic leakage in cells treated with CER+Q-VD-OPh (black arrows). Nec-1 pretreatment prevented the CER+Q-VD-OPh-induced changes

To examine CER-induced activation of necroptosis in a more physiologically relevant model, we isolated primary cytotrophoblasts cells from term placentae. Similarly to JEG3 cells, CER and CER+Q-VD-OPh treatments led to a significant increase in p-MLKL, which could be abolished by Nec-1 treatment (Figure 6a). TEM analysis of primary isolated trophoblasts demonstrated that CER+Q-VD-OPh treatment induced striking morphological alterations indicative of necroptotic cell death, including a translucent cytoplasm, loss of plasma membrane integrity and nuclear disruption with poorly defined chromatin (Figure 6c). Blinded scoring of trophoblast cell morphology based on the aforementioned criteria confirmed a statistically significant change in necrotic appearance of CER+Q-VD-OPh relative to control cells (Figure 6b). The elevated necrotic score of primary isolated trophoblasts following CER and caspase inhibition was primarily attributed to cytoplasmic translucence, membrane disruptions and chromatin decondensation, whereas the main morphological alteration identified in CER-treated cells was plasma membrane disruption (Supplementary Table 2). Finally, Nec-1 provided a powerful protection from CER+Q-VD-OPh-induced necroptosis in primary trophoblast cells, and resulted in a morphology resembling healthy control cells, furthering the evidence that trophoblasts specifically undergo necroptotic cell death under CER overload and caspase inhibitory conditions (Figures 6b and c, and Supplementary Table 2).

### Necroptosis activation is associated with aberrant trophoblast cell fusion

**Figure 7:**
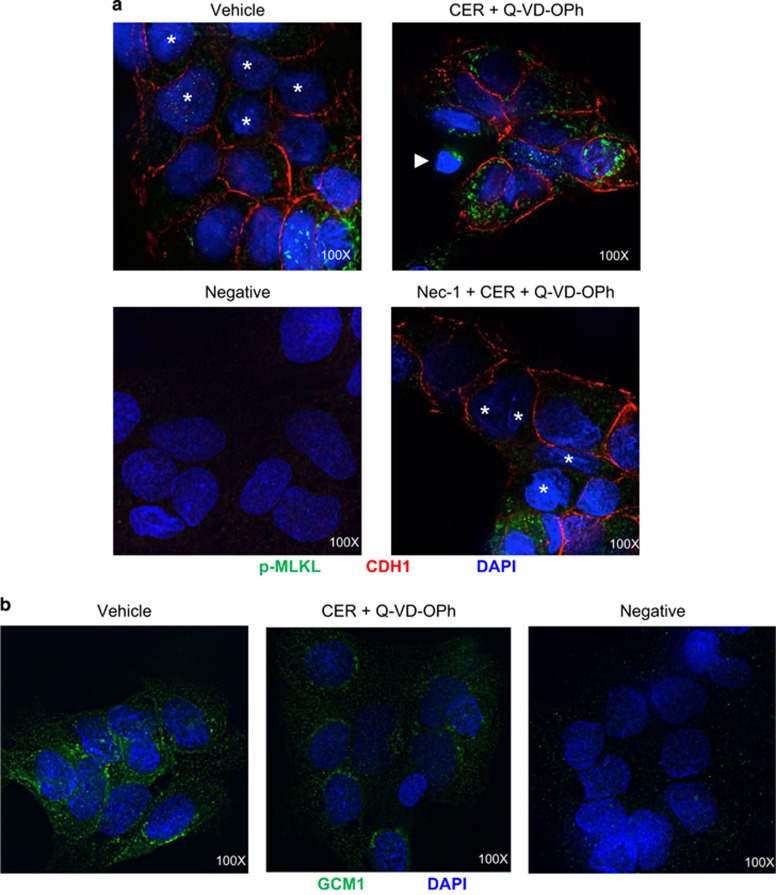
Necroptotic conditions impair trophoblast cell fusion. (**a**) Representative immunofluorescence images for p-MLKL (green) and CDH1 (red) in primary trophoblasts following exposure to 50 *μ*M CER+Q-VD-OPh in the presence or absence of Nec-1. Asterisks designate fusing cell nuclei. Twenty-four-hour exposure to 50 *μ*M CER+Q-VD-OPh led to increased expression of p-MLKL and retention of cell boundaries in primary trophoblast cells. A dying cell with shrunken nuclei is indicated by a white arrow and exhibits strong expression of p-MLKL punctae. Nuclei were counterstained with DAPI (blue). Rabbit and mouse IgG were used for the negative control. (**b**) Representative immunofluorescence images for GCM1 (green) in primary trophoblast cells subjected to 50 *μ*M CER+Q-VD-OPh treatment. CER+Q-VD-OPh led to a reduction in GCM1 expression compared with vehicle control. Nuclei were counterstained with DAPI (blue). Rabbit IgG was used as a negative control. Images shown at × 100 magnification

*In situ*, cytotrophoblasts undertake a tightly regulated differentiation process whereby they differentiate, lose cell boundaries and fuse with the overlying syncytium. Primary trophoblast cells represent a useful model for studying fusion as they fuse spontaneously in culture. The cell–cell adhesion protein E-cadherin (CDH1) is expressed in the plasma membrane of cytotrophoblasts but not syncytiotrophoblasts, and loss of CDH1-stained cell boundaries between neighboring cells is an indicator of trophoblast fusion.^22^ To examine the impact of necroptosis on trophoblasts fusion, we performed immunofluorescence co-staining for CDH1 and p-MLKL in primary trophoblast cells treated with CER+Q-VD-OPh with and without Nec-1 pretreatment. Although vehicle cells displayed patches of fused cells without CDH1 staining, primary CER+Q-VD-OPh-treated trophoblast cells retained cellular boundaries as indicated by CDH1-positive signal (Figure 7a) and exhibited strong p-MLKL expression, demonstrating activation of the necroptotic pathway. Cells in the process of dying as evidenced by their abnormal nuclei stained with DAPI were also found to express p-MLKL as discrete punctae. Cytotrophoblast fusion ability was recovered in Nec-1-treated cells, which displayed partial loss of CDH1-stained intercellular boundaries and reduced p-MLKL expression (Figure 7a).

Glial cell missing-1 (GCM1) is a transcription factor that is expressed in cytotrophoblasts before fusion and is downregulated in preeclamptic placentae.^23, 24^ Immunofluorescence staining revealed a decrease in GCM1 expression following CER+Q-VD-OPh treatment when compared with vehicle control, further supporting the notion of disrupted fusogenic properties in trophoblast cells (Figure 7b). Hence, conditions that favor necroptosis may interfere with cytotrophoblast fusion.

## Discussion

Preeclamptic pregnancies are characterized by CER overload, which heightens the rate of trophoblast cell death and turnover thereby contributing to the maternal endothelial dysfunction typical of this pathology.^16^ Herein, we report for the first time that human trophoblast cells utilize necroptosis as an alternative means of programmed cell death. In particular, we found that CER triggers the necroptotic machinery, primarily under conditions of caspase inhibition. Moreover, we provide the first evidence that preeclamptic placentae have an increased susceptibility to necroptosis compared with normotensive controls. We further establish that necroptosis interferes with normal trophoblast cell fusion, thereby contributing to aberrant syncytiotrophoblast cell turnover, characteristic of preeclampsia.

The placenta is critical to ensure growth and viability between embryonic days 9–12 in mice, and placental defects occurring during this period typically account for embryonic death. Interestingly, embryonic lethality of caspase-8 knockout mice occurs around embryonic days 10.5–11.5 and this is mediated by RIP3, a critical kinase comprising the necrosome complex.^25, 26^ However, the authors did not comment on whether there was a placental phenotype. Nevertheless, these findings point to a potential role for necroptosis in placental development.

Prior work has established that trophoblasts do not undergo necroptotic cell death in response to cytosolic double-stranded DNA, a stimulus that mimics conditions of bacterial infection.^27^ In contrast, our *in vitro* data using primary trophoblasts and choriocarcinoma JEG3 cells convincingly demonstrate that trophoblast cells are susceptible to necroptosis under conditions characterized by CER accumulation and caspase inactivation. Hence, trophoblasts may utilize necroptosis as a means of cell death, but this is dependent on the specific pro-death stimuli.

Our work establishes CER as a powerful inducer of necroptosis when combined with caspase inhibition. Cell death that is (1) independent of caspase-8 activity with morphological features of necrosis, (2) that demonstrates necroptotic protein expression and (3) that can be rescued using Nec-1, together provide convincing evidence for necroptosis.^1^ Both primary isolated trophoblasts and JEG3 cells exhibited all of these features when exposed to pharmacological caspase inhibition in conjunction with CER treatment *in vitro.* In addition, we found that knockdown of RIP3 and MLKL protected JEG3 cells from CER-induced necroptosis. Our findings of increased RIP1 expression and activated MLKL demonstrate for the first time a direct function of synthetic 16:0 CER on necroptosis signaling, and are in line with previous work showing that TNF as well as TRAIL induce caspase-independent death in a variety of cell lines by increasing intracellular CER levels.^28, 29^ In support of a role for CER in the induction of necroptosis, lipidomic analysis in human lymphoma U937 cells treated with TNF and the caspase inhibitor z-VAD-fmk revealed increased 16:0 CER compared with necrosis-resistant U937 cell lines.^30^ CER breakdown by overexpression of acid ceramidase protected L929 fibroblasts from TNF-induced cell death that was devoid of caspase activity, but addition of 2-OE to suppress acid ceramidase function, restored their sensitivity to TNF.^31^ Furthermore, studies from others demonstrated that restoring a balance to sphingolipid metabolism by interfering with CER rate-limiting enzymes confers protection from necroptotic cell death in L929 fibrosarcoma cells.^28, 29, 32^ Interestingly, RIP1 and CER were shown to act within the same pathway to induce caspase-independent programmed cell death as RIP1-deficient Jurkat cells did not accumulate CER and were resistant to death.^28^ RIP proteins have also been reported to be essential for reactive oxygen species (ROS) accumulation in TNF-induced necrotic cell death in monocytic and L929 cells.^33, 34^ ROS involvement in CER-mediated trophoblast necroptosis is supported by our published observation of oxidative stress-induced CER production^16^ and our current data indicating that exposure of JEG3 cells to sodium nitroprusside, a nitric oxide donor, significantly increased RIP1 expression (Supplementary Figure S1b). In addition, L929 cells treated with the omega-3 polyunsaturated fatty acid docosahexanoic acid were resistant to necroptosis because of attenuation of oxidative stress and CER production.^35^ CER may thus prove to be a useful target for preventing necroptosis in animal models and human diseases.

MLKL cellular localization, phosphorylation and oligomerization are critical when establishing necroptosis activation. Distinct clusters of p-MLKL have been observed in L929 fibroblast cells undergoing necroptosis, where p-MLKL was shown to form homo-oligomers before disruption of the cell membrane.^7, 10, 36^ These findings are consistent with our data demonstrating punctate expression of active p-MLKL following necroptosis induction with CER and Q-VD-OPh in both primary trophoblasts and JEG3 cells. In addition, we found a predominance of p-MLKL oligomers of approximately 250 kDa in the membrane-enriched fraction and expression of monomeric p-MLKL in the cytosol, which corroborate previous findings of a similar sized molecular weight banding pattern in HT-29 cells following TNF, Smac mimetic and z-VAD-fmk treatments.^10^

Recent advances in our understanding of the molecular mechanisms responsible for necroptosis demonstrate the feasibility of targeting it for the treatment of diseases that involve necrotic cell death and inflammation. Several inhibitors of necroptosis have been developed, including Nec-1, a RIP1 kinase inhibitor that has shown promise for mitigating pathological effects of necroptotic cell death *in vitro* and *in vivo* models,^11, 37, 38, 39, 40^ pointing to a key role for RIP1 in this process across organ systems. The potential of necrosome protein inhibitors as therapeutic agents is bolstered by the fact that RIP1, RIP3 and MLKL have few known functions besides necroptotic signaling, thereby minimizing the risk of off-target effects. Our findings demonstrate that inhibition of RIP1 kinase activity using Nec-1 protects trophoblast cells from CER+Q-VD-OPh-induced necroptosis by preventing MLKL phosphorylation and the formation of p-MLKL oligomers at the cell membrane, thereby maintaining trophoblast cell homeostasis.

Necroptosis has been linked to ischemia–reperfusion injury and pathologies characterized by an inflammatory response.^41, 42, 43, 44, 45^ Similarly, preeclampsia is characterized by placental hypoxia and oxidative stress.^46^ Herein, we identified increased expression of necrosome proteins in preeclamptic placentae. In addition, TEM analysis revealed that necroptotic events were present in cytotrophoblasts from preeclamptic pregnancies. There is evidence to support the increase in apoptosis^47^ and autophagy^16^ under high CER levels in the placenta. However, necroptosis is favored when caspases are inhibited or become defective; therefore, our finding of reduced caspase-8 activity in E-PE placentae supports the notion of increased trophoblasts susceptibility to necroptotic cell death in this pathological scenario. Importantly, necrotic trophoblast cells were visualized by TEM as sporadic events, and we propose necroptosis as an alternative mode of cell death to apoptosis and autophagy depending on the environmental conditions and needs of the cell.

Increasingly, E-PE and L-PE are categorized as preeclamptic diseases with differing etiologies despite having similar presenting features,^48, 49^ as E-PE originates from defective placentation dependent on disrupted oxygen-sensing mechanisms, whereas L-PE is linked to inflammatory alterations and maternal constitutional factors.^50, 51, 52^ In this study, placentae from pregnancies complicated by both E-PE and L-PE exhibited augmented necrosome protein expression and activation. However, we did not detect any deficiencies in caspase-8 activity in L-PE cases, suggesting that necroptosis may be especially favored in E-PE. We surmise that necroptosis may contribute to both subtypes, but is stimulated by oxidative stress in E-PE, whereas it results from cellular senescence and aging in L-PE.

Finally, exploring the biological relevance of necroptosis on placentation led us to hypothesize that necroptosis may interfere with trophoblast differentiation processes. Although predominantly known as an initiator caspase in apoptosis, caspase-8 exhibits non-apoptotic functions and is required for syncytial fusion of cytotrophoblast cells.^53^ Blockage of capase-8 activity has been proposed to halt fusion and prevent trophoblast cell turnover by apoptosis, thereby leading to cell death via necrosis;^53^ however, necroptosis was not examined. The data presented in this study demonstrate that primary trophoblast cells treated with CER+Q-VD-OPh retained cell boundaries, an index of impaired fusion, and exhibited decreased expression of fusogenic GCM1 protein. Thus, conditions that favor necroptosis, namely inactive caspase-8, could disrupt cytotrophoblast differentiation toward fusion. It is established that preeclamptic placentae are characterized by dysregulated cell fusion processes and reduced GCM1 expression.^24, 54^ Hence, necroptotic events may not only contribute to the excessive cell death that is observed in preeclampsia, but may also disrupt the normal trophoblast fusion. Whether necroptosis-targeted cells die before fusion thereby impairing syncytium formation, or cells with impaired fusion machinery are selectively eliminated by necroptosis remains to be established.

**Figure 8:**
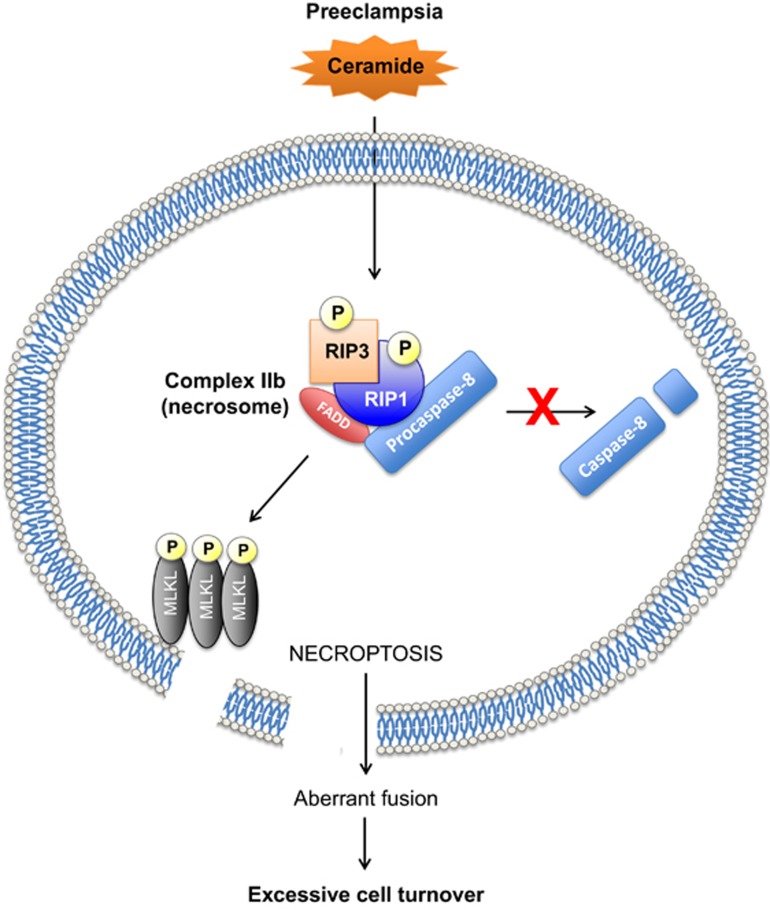
Putative model of CER-mediated necroptosis and its role in the pathogenesis of preeclampsia. CER overload in preeclamptic placentae primes RIP1/RIP3 necrosome assembly. Conditions of reduced caspase-8 activity, as demonstrated in E-PE, favor CER to cause MLKL phosphorylation and oligomerization at the cell membrane where it executes necroptosis by permeabilizing the membrane. Necroptotic cell death also prevents normal cytotrophoblast fusion that is required to replenish the syncytiotrophoblast layer, resulting in improper cell turnover

In summary, we propose a putative model of necroptosis in preeclampsia (Figure 8). The excessive CER content in preeclamptic placentae primes RIP1/RIP3 necrosome assembly, which under conditions of caspase-8 deficiency, as observed in severe E-PE cases, triggers MLKL phosphorylation. Subsequently, p-MLKL oligomerization at the plasma membrane interferes with normal cytotrophoblast fusion processes and executes necroptosis. As a result, the syncytium is not adequately refurbished to ensure its proper function and sheds in excess, exacerbating the maternal clinical manifestations. Uncovering the molecular mechanisms that contribute to excessive placental cell death in preeclampsia can identify new avenues of therapeutic intervention for this currently untreatable pregnancy disorder. Given the relative specificity and drugability of the proteins in the necroptosis pathway, future investigations aimed at targeting their function in the human placenta are warranted.

## Materials and methods

### Tissue collection

Informed consent was obtained from patients before collection of tissue samples. All procedures were conducted according to Ethics Guidelines outlined by the University of Toronto Faculty of Medicine, Research Ethics Board of Mount Sinai Hospital, and the World Medical Association Declaration of Helsinki. Preeclamptic placentae were selected on the basis of American College of Obstetrics and Gynecology clinical and pathological criteria.^21^ Placentae from normotensive age-matched deliveries without complications, infections or signs of disease were used as controls. The clinical characteristics of the patients are outlined in Supplementary Table 1. All placental specimens were obtained immediately after delivery, and either snap frozen in liquid nitrogen or fixed in 4% paraformaldehyde until further analysis.

### Cell line culture and treatments

Human choriocarcinoma JEG3 cells (ATCC, Manassas, VA, USA) were cultured in Eagle's minimal essential medium (EMEM) supplemented with 10% (vol/vol) fetal bovine serum (FBS) and 1% (10 000 units/ml) of pen/strep at 37 °C under standard culture conditions (5% CO_2_, 21% O_2_). Q-VD-OPh hydrate (ApexBio Technology LLC, Houston, TX, USA) was reconstituted in dimethyl sulfoxide (DMSO) before treatment of cells at a concentration of 50 *μ*M. C16:0 CER (Enzo Life Sciences, Farmingdale, NY, USA) was dissolved in anhydrous ethanol and used at a final concentration of 50 *μ*M, whereas 2-OE (Invitrogen, Carlsbad, CA, USA) was diluted in DMSO at a concentration of 25 *μ*M. Nec-1 (Cayman Chemical Company, Ann Arbor, MI, USA) was also diluted in DMSO and added to media at a concentration of 25 *μ*M 1 h before CER and Q-VD-OPh treatment, to block necroptotic signaling.

### Primary trophoblast cell isolation

Primary trophoblast cell isolation was performed as previously described. ^55, 56^ Placentae (*n*=7) were obtained after elective cesarean sections from uncomplicated term pregnancies. Briefly, placental tissue was dissected to remove calcifications and blood clots, and digested with 0.05 mM trypsin (GIBCO 27250-018; Invitrogen) and 0.008 mM DNase I (SIGMA DN25; Sigma-Aldrich Corp. St. Louis, MO, USA) at 37 °C in Dulbecco's modified Eagle's medium (DMEM; Invitrogen). The obtained cell suspension was subsequently passed through a 70 *μ*M nylon sieve (Becton, Dickson and Company, Franklin Lakes, NJ, USA), and layered on a discontinuous 5–70% (vol/vol) Percoll (GE Healthcare, Little Chalfont, UK) gradient. Following centrifugation, the intermediate layer containing cytotrophoblasts was removed and washed with DMEM. Cells were plated at 2 × 10^6^ cells in 4-ml cell culture dishes in complete DMEM containing 10% FBS and 10 000 U/ml of penicillin and streptomycin at 37 °C and maintained at (8% O_2_, 5% CO_2_). Cell purity was assessed by immunofluorescence staining for cytokeratin-7, an epithelial cell marker. After 24 h, cells were treated as per experimental design and collected in radioimmunoprecipitation assay (RIPA) buffer for WB analysis or fixed in 4% (vol/vol) paraformaldehyde for immunofluorescence staining.

### Western blotting and immunoprecipitation

Placental tissue was homogenized in RIPA buffer supplemented with protease inhibitor cocktail (Roche Diagnostics, Indianapolis, IN, USA). Following their respective treatment conditions, primary and JEG3 cells were washed once with PBS and collected in RIPA buffer. Western blotting was performed as previously described using lysates from PTC, preeclampsia and term placentae, JEG3 or primary trophoblast cells.^16^ Briefly, 50 *μ*g of protein were subjected to SDS-PAGE and then transferred to a polyvinylidene difluoride membrane. The membranes were blocked with 5% (wt/vol) skimmed milk powder in Tris-buffered saline containing 0.1% (vol/vol) Tween-20 (TBST) for 1 h at room temperature before overnight incubation with primary antibodies at 4 °C. Membranes were next washed in TBST and incubated with secondary antibody for 1 h. After washing with TBST, blots were visualized by enhanced chemiluminescence reagent (PerkinElmer, Life Sciences, Waltham, MA, USA). In all cases, *β*-actin (ACTB) was used as a loading control.

Immunoprecipitation experiments were performed as described previously.^17^ Anti-RIP3 antibodies (1.2 *μ*g) were added to 300 *μ*g of total cell extract (in 300 *μ*l) in RIPA buffer and incubated at 4 °C on a rocker overnight. Protein G agarose was added to the samples and incubation continued for 2 h. Immunocomplexes were collected by centrifugation, washed successively with PBS and RIPA buffer and dissolved in SDS sample buffer. Samples were then subjected to SDS-PAGE as described above and immunoblotted for RIP1 and RIP3.

### Flow cytometry

JEG3 cells treated with CER+Q-VD-OPh were dissociated using 0.5 ml per well TrypLE express (Invitrogen), washed and processed in accordance with the FITC Annexin V Apoptosis Detection Kit 1 (BD Pharmingen, San Jose, CA, USA) at a concentration of 1 × 10^6^ cells/ml. In brief, Annexin V and/or propidium iodide were added to the cells and incubated alongside a binding buffer for 15 min at room temperature protected from light, then thoroughly washed. Data were acquired using the Beckman Coulter Gallios 10/3 flow cytometer and analyzed with Kaluza flow cytometry software (Beckman Coulter, Brea, CA, USA). Annexin V-positive events were measured using the FL-1 channel (525 emission maximum), whereas the FL-4 channel (670 nm emission maximum) was used for propidium iodide-positive events. Unstained cells, cells stained only with FITC Annexin V and cells stained only with propidium iodide were included as controls.

### Immunofluorescence

Immunofluorescence staining of cells was performed as previously described.^54^ Briefly, primary isolated trophoblasts and JEG3 cells sections were washed in PBS (pH 7.4) and fixed with 4% (vol/vol) paraformaldehyde for 15 min. Cells were permeabilized with Triton X-100, and blocked in 5% (vol/vol) normal donkey serum. Cells were first incubated with primary rabbit anti-human p-MLKL antibody (1:100) overnight, and later in Alexa Fluor 488-conjugated donkey anti-rabbit secondary antibody (1:200) (Invitrogen) for 1 h after washing in PBS. Samples were next stained with DAPI and mounted in Immu-Mount fluorescent mounting medium (Thermofisher Scientific, Waltham, MA, USA). Images were captured using a DeltaVision deconvolution microscope (Applied Precision, LLC, Issaquah, WA, USA).

### RIP3 and MLKL RNA interference knockdown

JEG3 cells were grown to 60% confluency in EMEM containing 10% (vol/vol) FBS in six-well plates. Four hours before transfection, media were replaced to FBS-free EMEM containing vehicle (EtOH+DMSO) or CER+Q-VD-OPh. MLKL silencer siRNA duplexes and silencer negative control scrambled siRNA sequences were obtained from Ambion, Inc. (Austin, TX, USA). Briefly, 30 nM MLKL siRNA was added to 200 *μ*l jetPRIME buffer (Polyplus transfection, Illkirch, France). Cells were collected 24 h after treatment for protein isolation and TEM analysis.

### Cell membrane isolation

Following 24-h treatment with vehicle (ethanol and DMSO), CER+Q-VD-OPh or Nec-1+CER+Q-VD-OPh, JEG3 cells were collected in a HEPES sucrose buffer and sonicated three times for 30 s, with 30-s rest on ice in between. Cells lysates were next spun at 720 × *g* for 10 min to pellet nuclei. The supernatant was collected and spun in an ultracentrifuge at 100 000 × *g* overnight at 4 °C. This pellet was resuspended in RIPA buffer and represents the membrane fraction. The cytoplasmic fraction (supernatant) was concentrated using Amicon centrifugal filter devices (EMD Millipore, Billerica, MA, USA) spun at 4 °C at 4000 × *g* for 45 min, and samples were then prepared for western blotting. As an alternative method, JEG3 cells were collected in a Triton X-114 lysis buffer and subjected to phase separation as previously described.^10^

### Antibodies

The following antibodies were purchased from Santa Cruz Biotechnology, Dallas, TX, USA: cleaved caspase-8 (sc-1226), RIP3 (sc-47368), ACTB (sc-1616) and *α*-tubulin (TUBA; sc-31779). Cleaved caspase-3 (no. 9661) and RIP1 (no. 3493) were purchased from Cell Signaling Technology, Danvers, MA, USA. Antibodies specific to PLAP (ab118856), RIP3 (ab56164), total MLKL (ab194699), phospho-MLKL (ab187091 and its competing peptide ab206929), cytokeratin-7 (ab9021), and E-cadherin (ab1416) were purchased from Abcam, Cambridge, UK. GCM1 antibody (P100836_P050) was purchased from Aviva Systems Biology, Corp., San Diego, CA, USA. Secondary antibodies include horseradish peroxidase-conjugated donkey anti-goat, goat anti-rabbit and goat anti-mouse from Santa Cruz Biotechnology. For immunofluorescence, the following secondary antibodies were used: Alexa Fluor 594 donkey anti-rabbit and Alexa Fluor 488 donkey anti-rabbit from Life Technologies, Carlsbad, CA, USA.

### Caspase-8 activity assay

Caspase activity was measured using the Caspase-8 Fluorometric Assay Kit (Abcam ab39534) according to the manufacturer's instructions. Homogenized tissue lysates from PTC (*n*=6) and E-PE (*n*=6) were run in duplicate for fluorescence measurement in a 96-well plate (M200 infinite plate reader, Tecan, Männedorf, CH, Switzerland).

### Transmission electron microscopy

Preeclamptic (*n*=8) and control placentae (*n*=7) were collected and fixed with 2.5% gluteraldehyde in 0.1 m phosphate buffer for 24 h at 4 °C within 10 min of delivery. Primary trophoblast cells were seeded at a density of 1 × 10^6^ cells per well on coverslips pretreated with 10% FBS. The cells were allowed to attach overnight, then treated with 50 *μ*M C16:0 CER, and/or 50 *μ*M Q-VD-OPh, with or without 25 *μ*M Nec-1 under normoxic conditions (8% O_2_ for primary cells, 20% O_2_ for JEG3) at 37 °C for 24 h. Samples were fixed in 2% (vol/vol) glutaraldehyde in 0.1 M sodium cacodylate buffer pH 7.3 for 1 h at room temperature. Tissues and cell samples were cut into ultrathin sections and imaged using a Tecnai 20 transmission electron microscope (FEI, Hillsboro, OR, USA). TEM images of primary cytotrophoblasts were randomly selected from each treatment condition (vehicle *n*=12, CER *n*=13, CER+Q-VD-OPh *n*=13 and Nec-1+CER+Q-VD-OPh *n*=13) and subjected to quantification of necroptotic morphological characteristics using the following blinded scoring method. Three blinded assessors were asked to score TEM images based on the following criteria characteristic of necrotic cells: mitochondrial swelling, translucent cytoplasm, decondensed chromatin and/or loss of plasma membrane integrity. A score of 1 was assigned to each feature, which was readily observable, and 0 if absent. When scoring for a particular criterion differed between assessors, the majority count was deemed to be the consensus, generating a ‘corrected score'. The corrected scores from each criterion were totaled per cell, and averaged along with cells from the same treatment condition.

### Statistical analysis

Densitometric analysis was carried out using ImageQuant 5.0 (Molecular Dynamics, GE Healthcare, La Jolla, CA, USA) and statistical differences were analyzed using GraphPad Prism 7.0 software, Bio-Science AB, Bjorkgatan, Uppsala, Sweden using Student's *t*-test or one-way ANOVA where applicable. Data are expressed as the mean±S.E.M. and a value of *P*<0.05 was considered statistically significant (*P<0.05, **P<0.01 and ***P<0.001). Cronbach's alpha was used to determine the internal consistency of our blinded TEM scoring test.
