# Supplementary material for: Augmented trophoblast cell death in preeclampsia can proceed via ceramide-mediated necroptosis
Source: Cell Death Dis. 2017 Feb 2;8(2):e2590–. doi: 10.1038/cddis.2016.483 (PMC5386461; doi:10.1038/cddis.2016.483)
Supplement: Supplementary Figures [file cddis2016483x1.docx]

**SUPPLEMENTARY FIGURES LEGENDS**

**Supplementary Figure 1. (a)** Western Blot for p-MLKL in JEG3 and primary isolated trophoblast cells after CER+Q-VD-Oph treatment probed with p-MLKL antibody (left) and p-MLKL antibody + competing peptide (right) **(b)** JEG3 cells treated with 2.5 mM sodium nitroprusside (SNP) for 24 hours show a significant elevation in RIP1 protein levels, but not in RIP3. Protein levels were normalized to ACTB and densitometric quantification is expressed as fold change relative to vehicle control.

**Supplementary Figure 2.** (**a**) Densitometric analysis of p-MLKL and RIP1 protein expression levels in placentae from labour and no labour deliveries, (**b**) C-section versus spontaneous vaginal deliveries, and (**c**) from pregnancies carrying female or male fetuses.
