# Supplementary material for: Augmented trophoblast cell death in preeclampsia can proceed via ceramide-mediated necroptosis
Source: Cell Death Dis. 2017 Feb 2;8(2):e2590–. doi: 10.1038/cddis.2016.483 (PMC5386461; doi:10.1038/cddis.2016.483)
Supplement: Supplementary Information [file cddis2016483x2.pdf]

Table 1. Clinical parameters of the study population.

Clinical Parameters	Preterm controls n=18	Early-onset preeclampsia n=37	Late-onset preeclampsia n=22	Term controls n=32
Gestational age at delivery (weeks)	29.5±0.6	28.5±0.5	37.3±0.4	39.0±0.2
Fetal Weight (g)	1439±168.4	1038±96.2 *	2921±257.4	3467.8±141.4
Fetal Sex	33.3% F, 66.7% M	35.7% F, 64.3% M	11.1% F, 88.8% M	35% F, 65% M
Blood Pressure (mm Hg)	S: 114.3±4.8<br>D: 69.9±3.3	S:168.3±4.4 ***<br>D: 102.6±3.2 ***	S:151.6±9.2 *<br>D: 94.0±5.8 *	S:117.5±2.9<br>D: 69.5±0.6
Proteinuria (g/day)	absent	3.7±0.17	2.25±0.75	absent
Mode of Delivery	33% CS<br>67% VD	93.7% CS<br>6.3% VD	77.8% CS,<br>22.2% VD	94.7% CS<br>5.3% VD

F, female; M, male;
S, systolic; D, diastolic;
CS, caesarian section; VD, vaginal delivery.
*p<0.05; ***p<0.001

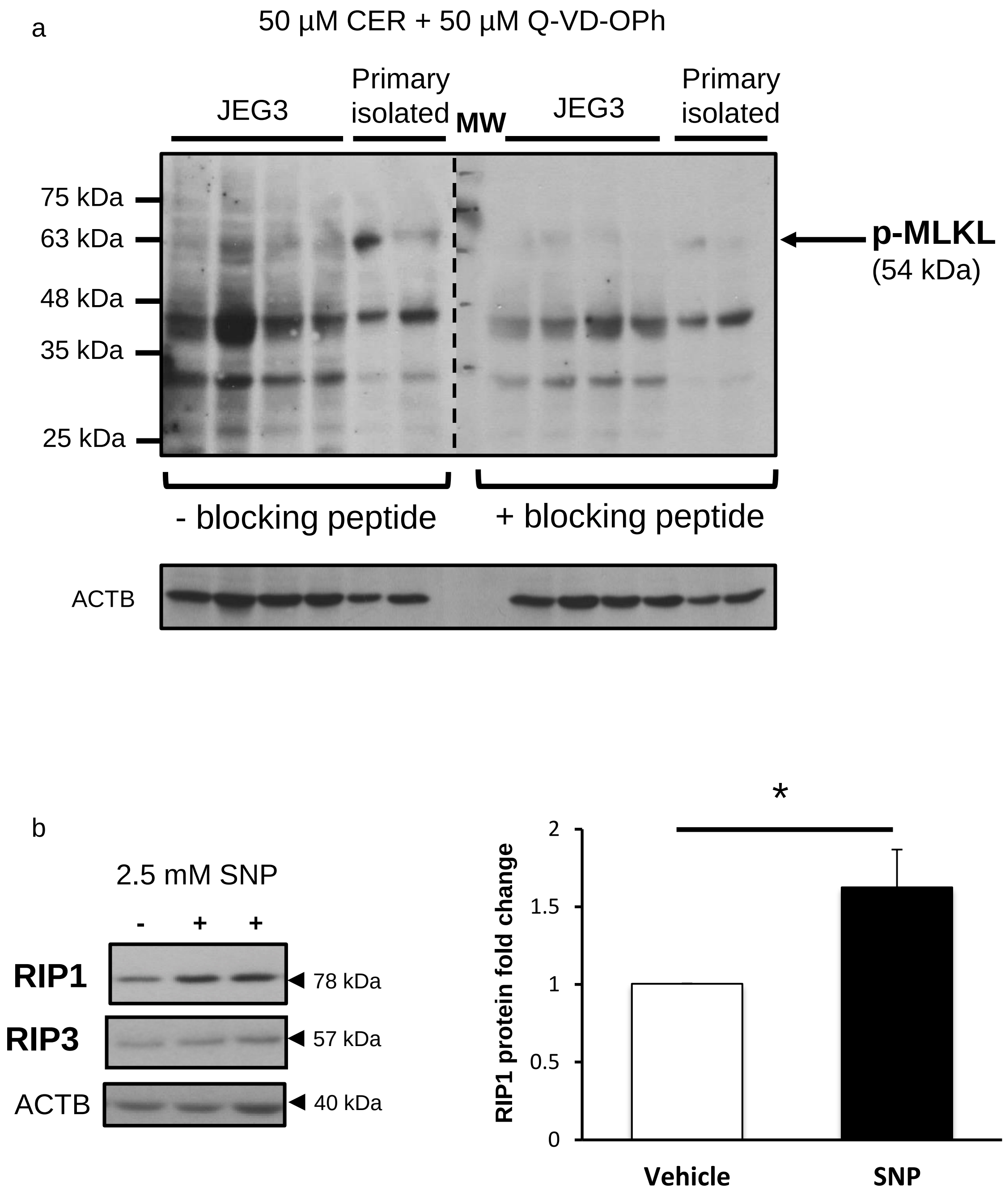

Supplementary Figure 1

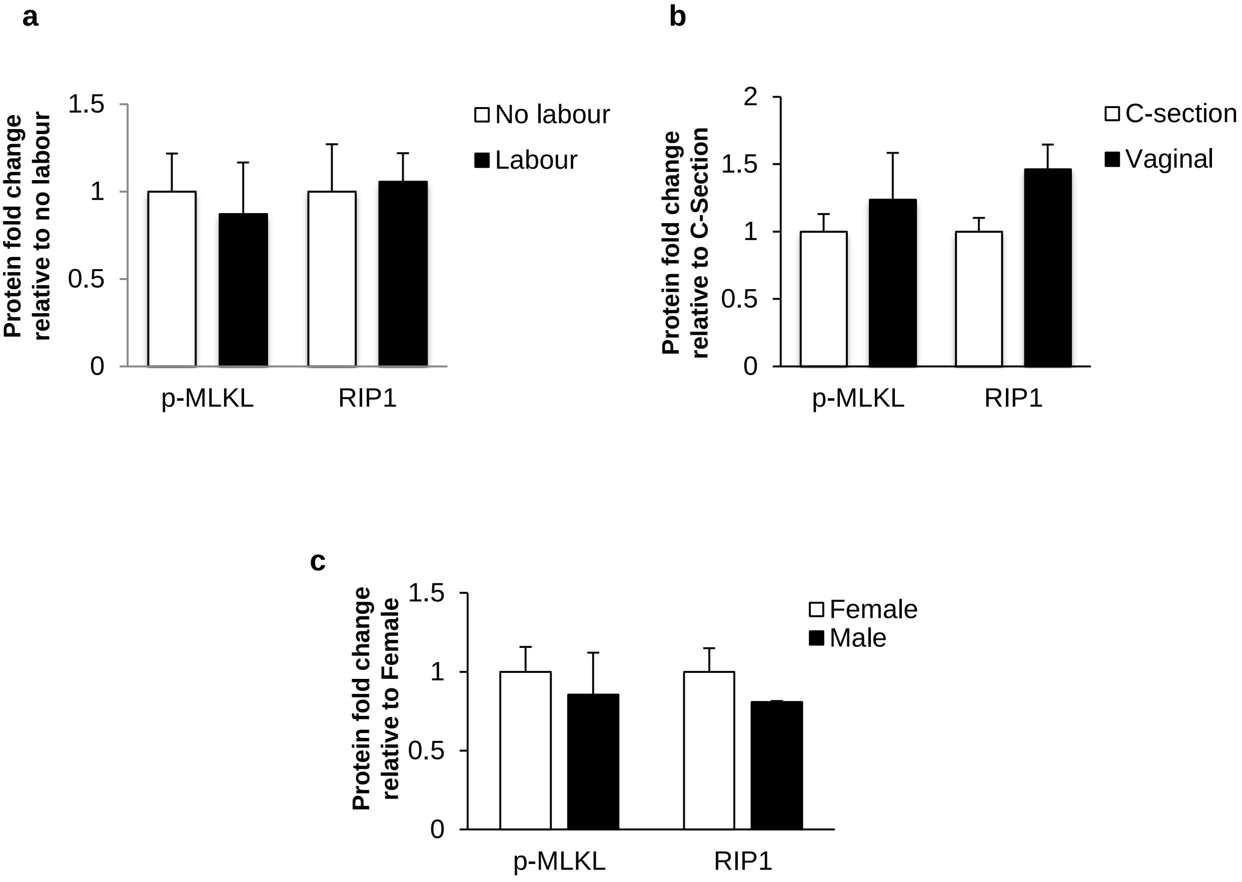

Supplementary Figure 2

Table 2. Analysis of necrotic morphological criteria in primary isolated cells by TEM

	Vehicle (n=12)	Ceramide (n=13)	CER+Q-VD-OPh (n=13)	Nec-1+CER+ Q-VD-OPh (n=12)
Less dense cytoplasm	8%	15%	77%	0%
Loss of membrane	25%	69%	77%	17%
Decondensed chromatin	8%	23%	85%	0%
Swollen mitochondria	8%	8%	23%	17%
